# Bis{2-meth­oxy-6-[(3-pyrid­yl)methyl­imino­meth­yl]phenolato}copper(II)

**DOI:** 10.1107/S1600536809029523

**Published:** 2009-08-12

**Authors:** Yun Wang, Li-Li Zhu, Bai-Wang Sun

**Affiliations:** a Department of Chemistry, Key Laboratory of Medicinal Chemistry for Natural Resources, Ministry of Education, Yunnan University, Kunming 650091, People’s Republic of China; b Ordered Matter Science Research Center, College of Chemistry and Chemical Engineering, Southeast University, Nanjing 210096, People’s Republic of China

## Abstract

In the mononuclear title complex, [Cu(C_14_H_13_N_2_O_2_)_2_], the Cu^II^ atom lies on an inversion centre and adopts a square-planar coordination geometry. The dihedral angle formed by the pyridine and benzene rings is 74.61 (5)°. Intra­molecular C—H⋯O hydrogen bonds are present. The crystal structure is stabilized by weak aromatic π–π stacking inter­actions involving neighbouring pyridine rings [centroid–centroid distance = 3.853 (2) Å].

## Related literature

For a related structure, see: Wang *et al.* (2008 ▶). For the synthetic procedure, see: Kannappan *et al.* (2005 ▶); Zhao *et al.* (2008 ▶).
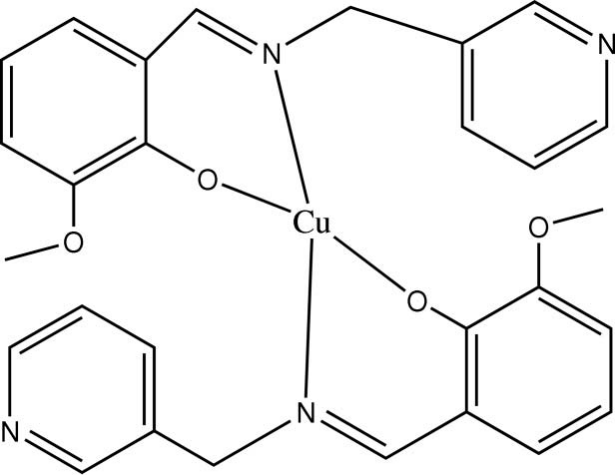


## Experimental

### 

#### Crystal data

[Cu(C_14_H_13_N_2_O_2_)_2_]
*M* *_r_* = 546.07
Monoclinic, 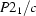

*a* = 11.455 (2) Å
*b* = 14.414 (3) Å
*c* = 7.5491 (15) Å
β = 102.55 (3)°
*V* = 1216.7 (4) Å^3^
*Z* = 2
Mo *K*α radiation
μ = 0.94 mm^−1^
*T* = 293 K
0.20 × 0.20 × 0.20 mm

#### Data collection

Rigaku SCXmini diffractometer
Absorption correction: multi-scan (*CrystalClear*; Rigaku, 2005 ▶) *T*_min_ = 0.824, *T*_max_ = 0.828
11771 measured reflections
2667 independent reflections
2430 reflections with *I* > 2σ(*I*)
*R*_int_ = 0.035

#### Refinement

*R*[*F* ^2^ > 2σ(*F* ^2^)] = 0.035
*wR*(*F* ^2^) = 0.102
*S* = 1.41
2667 reflections
169 parameters
H-atom parameters constrained
Δρ_max_ = 0.25 e Å^−3^
Δρ_min_ = −0.62 e Å^−3^

### 

Data collection: *CrystalClear* (Rigaku, 2005 ▶); cell refinement: *CrystalClear*; data reduction: *CrystalClear*; program(s) used to solve structure: *SHELXS97* (Sheldrick, 2008 ▶); program(s) used to refine structure: *SHELXL97* (Sheldrick, 2008 ▶); molecular graphics: *SHELXTL/PC* (Sheldrick, 2008 ▶); software used to prepare material for publication: *SHELXTL/PC*.

## Supplementary Material

Crystal structure: contains datablocks I, global. DOI: 10.1107/S1600536809029523/rz2354sup1.cif

Structure factors: contains datablocks I. DOI: 10.1107/S1600536809029523/rz2354Isup2.hkl

## Figures and Tables

**Table 1:** Hydrogen-bond geometry (Å, °)

*D*—H⋯*A*	*D*—H	H⋯*A*	*D*⋯*A*	*D*—H⋯*A*
C9—H9*B*⋯O2^i^	0.97	2.28	2.862 (2)	118

Symmetry code: (i) 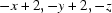.
